# Transparent Sol-Gel Oxyfluoride Glass-Ceramics with High Crystalline Fraction and Study of RE Incorporation

**DOI:** 10.3390/nano9040530

**Published:** 2019-04-03

**Authors:** Giulio Gorni, Jose J. Velázquez, Jadra Mosa, Glenn C. Mather, Aida Serrano, María Vila, Germán R. Castro, David Bravo, Rolindes Balda, Joaquín Fernández, Alicia Durán, Yolanda Castro

**Affiliations:** 1 Instituto de Cerámica y Vidrio, CSIC, 28049 Madrid, Spain; ggorni@icv.csic.es (G.G.); jmosa@icv.csic.es (J.M.); mather@icv.csic.es (G.C.M.); aida.serrano@icv.csic.es (A.S.); aduran@icv.csic.es (A.D.); 2 FunGlass—Centre for Functional and Surface Functionalized Glass, Alexander Dubček University of Trenčín, Trenčín 91150, Slovakia; jose.velazquez@tnuni.sk; 3 SpLine, Spanish CRG Beamline—European Synchrotron Radiation Facility (ESRF), 38043 Grenoble, France; maria.vila-santos@esrf.fr (M.V.); german.castro@esrf.fr (G.R.C.); 4 Instituto de Ciencia de Materiales de Madrid, CSIC, 28049 Madrid, Spain; 5 Departamento Física de Materiales, Facultad de Ciencias, Universidad Autónoma de Madrid (UAM), 28049 Madrid, Spain; david.bravo@uam.es; 6 Departamento Física Aplica I, Escuela Superior de Ingeniería, Universidad del País Vasco (UPV-EHU), 48013 Bilbao, Spain; rolindes.balda@ehu.eus; 7 Centro de Física de Materiales, (UPV/EHU-CSIC), 20018 San Sebastian, Spain; 8 Donostia International Physics Center (DIPC), 20018 San Sebastian, Spain; xuaco@dipc.org

**Keywords:** glass-ceramic, oxyfluoride, rare-earth, crystallization, sol-gel

## Abstract

Transparent oxyfluoride glass-ceramic films and self-supported layers with composition 80SiO_2_-20LaF_3_ doped with Er^3+^ have been successfully synthesized by sol-gel process for the first time. Crack-free films and self-supported layer with a maximum thickness up to 1.4 µm were obtained after heat treatment at the low temperature of 550 °C for 1 min, resulting in a LaF_3_ crystal fraction of 18 wt%, as confirmed by quantitative Rietveld refinement. This is the highest value reported up to now for transparent oxyfluoride glass-ceramics prepared by sol-gel. This work provides a new synthesis strategy and opens the way to a wide range of potential applications of oxyfluoride glass-ceramics. The characterization by a wide range of techniques revealed the homogeneous precipitation of LaF_3_ nanocrystals into the glass matrix. X-ray absorption spectroscopy and electron paramagnetic resonance confirmed that the Er^3+^ ions are preferentially embedded in the low phonon-energy LaF_3_ nanocrystals. Moreover, photoluminescence (PL) measurements confirmed the incorporation of dopants in the LaF_3_ nanocrystals. The effective concentration of rare-earth ions in the LaF_3_ nanocrystals is also estimated by X-ray absorption spectroscopy.

## 1. Introduction

Oxyfluoride glass-ceramics (OxGCs) have attracted great interest in the field of photonics since the pioneering work of Wang and Ohwaki [1]. OxGCs are polycrystalline materials containing fluoride nano-crystals (NCs) that crystallize in a glass matrix during a controlled heat treatment of the precursor glass. Alumina-silicate glass matrices are suitable, due to their excellent mechanical, thermal and chemical properties compared to phosphate or fluoride glasses. In addition, OxGCs may host Rare-Earth (RE) ions in the low-phonon energy fluoride crystals (300–450 cm^−1^), thereby increasing the luminescence efficiency with respect to the corresponding oxide glasses [2,3,4,5,6,7,8,9,10,11,12,13,14]. The melt-quenching (MQ) method has been routinely used to prepare OxGCs that showed excellent optical properties and a significant enhancement of photoluminescence with respect to the precursor glasses. Moreover, the possibility to draw fibers and convert the core into a GC revealed the importance of this method for the preparation of novel devices with new or improved optical efficiencies [15,16,17,18,19,20]. However, the high melting temperatures (1400–1700 °C) greatly affect the fluoride content, limiting the crystal phase content to 10 wt% or less for some crystal phases, such as LaF_3_ [7,9,10]. Furthermore, phase separation and spontaneous fluoride crystallization are usually present, due to fluorine immiscibility at high temperature in oxide-glass matrices. For these reasons, this method still has to overcome some problems and efforts are required to improve the quality and processing of these materials. Another crucial point concerns the incorporation of dopants into the fluoride NCs. In fact, it is known that in melted glasses there is a portion of RE ions that remain outside the fluoride NCs even after heat treatment for prolonged periods [10,12]. This phenomenon is explained considering the high glass viscosity at typical crystallization temperatures (*T*_g_ + 20–100 °C) that limits ionic diffusion. Moreover, due to the depletion of crystal formers in the glass matrix, a viscous Si-enriched shell forms around the crystals, which further limits ionic diffusion. A challenging goal is to obtain homogeneous materials with much higher fluoride contents and with higher dopant incorporation in the NCs, thus improving the optical efficiency.

The sol-gel (SG) route appears to be a promising alternative to prepare OxGC films and bulk materials for different applications, such as planar waveguides or integrated optics [21,22,23,24,25,26,27,28,29,30,31,32,33,34,35,36]. Ballato et al. [35] proposed to use LaF_3_ into planar photonic devices, such as planar confined waveguide and Kumar et al. [36] described Er^3+^ doped LaF_3_ transparent gels for its use in the infrared (IR) waveguide amplifiers at 1.5 μm.

The SG process is a cheap and extremely flexible chemical method for obtaining pure and very homogenous materials, as well as offering a wide range of possible nano/micro-structures at relatively low sintering temperatures (100–600 °C). Synthesis classically involves the hydrolysis and polycondensation of metal salts or metal-organic precursors, such as tetraethyl orthosilicate (TEOS), in alcoholic media [37]. In the late nineties, Fujihara et al. prepared the first OxGC SG materials in a two-step process [21] involving preparation of silica sol using the classical route followed by mixing of fluoride and RE-ion precursors (acetates, nitrates, chlorides etc.) dissolved in ethanol or acidic medium. The mixing of both solutions, with subsequent controlled crystallization, leads to the precipitation of fluoride crystals in the silica-glass matrix. However, the literature mostly describes the preparation of OxGC materials with a nominal content of 5 mol% active crystal phase, and with the final crystal fraction not being estimated [38]. For example, Szpikowska-Sroka reported 3 wt% of the crystallized phase for a nominal concentration of 5 mol% [34] Such concentrations are even lower than those obtained by MQ [2,10,12]. Other authors have attempted to increase the crystal content up to 15 mol% but opaque materials were obtained [21]. Moreover, high treatment temperatures (800–1000 °C) are used to obtain OxGCs from the amorphous precursors, even though the typical crystallization temperature of fluorides is between 280–350 °C. To date, the use of sol-gel to obtain OxGCs has not been widely investigated because no significant improvement in structural and optical properties has been achieved since the earliest papers. There is still much work to be performed on correlating the crystallization mechanism of these materials with the structure, dopant incorporation and optical properties.

In this work, novel OxGC materials with a much higher active crystal phase content and sintered at a much lower temperature than previously reported have been prepared by taking advantage of the particular benefits of the SG process. In addition, it was demonstrated how this method leads to precipitation of RE-doped NCs using short heat treatment times. Moreover, it is shown how most RE ions are incorporated into the NCs during the crystallization process.

## 2. Materials and Methods

### 2.1. Materials Preparation

Sols of composition (mol%) 80SiO_2_-20LaF_3_ doped with 0.1 and 0.5 Er^3+^ were prepared by partially replacing TEOS (Sigma Aldrich, St. Louis, MO, USA) with methyl-trimethoxy-silane (MTES, abcr, Karlsruhe, Germany) as a precursor with a TEOS/MTES molar ratio of 40/60. The synthesis was performed in two- step process starting from a silica sol with molar ratio: 0.4TEOS:0.6MTES:2.5CH_3_CH_2_OH:1H_2_O(0.1HCl):0.2CH_3_COOH (Merck, Darmstadt, Germany) stirred for 2 h at room temperature. Subsequently, La(CH_3_COO)_3_ (Sigma Aldrich), ethanol (EtOH), H_2_O and trifluoroacetic acid (TFA, Sigma Aldrich) in the molar ratio 1:5:4:4 was stirred for 2 h at 40 °C and then mixed with the silica sol in the ratio 0.8(TEOS + MTES):0.2La.

Thin films on silica and silicon wafers (111) were prepared by dip coating using withdrawal rates of 10–35 cm/min. Finally, the films were treated at 550 °C for 1 min and 1 h using a heating rate of 10 °C/min.

Moreover, self-supported layers (bulk-like monoliths 1–2 mm thick) of 80SiO_2_-20LaF_3_ composition doped with 0.1 and 0.5 Er^3+^ were prepared with TEOS and TEOS-MTES using the molar ratio 1TEOS:3EtOH:2H_2_O(0.1HCl) and 0.4TEOS:0.6MTES:3EtOH:2H_2_O(0.1HCl), respectively. An acetate dissolution, similar to that used for thin films, was added, raising the H_2_O content to obtain a total molar ratio 1TEOS:10H_2_O or 1(TEOS+MTES):10H_2_O.

Then, the sol was deposited into Petri dishes, covered, sealed and kept at 50 °C for 1 week to obtain the xerogel samples. GC samples were obtained by heating the xerogels at 550 °C for 1 min up to 1 h, with an intermediate step at 150 °C to remove water and solvents. Samples prepared with only TEOS were treated up to 650 °C for 1 h using a heating rate of 1 °C/min. The samples will be labelled with the composition followed by the silica precursor used for their preparation (TEOS) or (TEOS-MTES).

### 2.2. Characterization of Coatings and Self-supported Layers

Thin films deposited onto a silica and silicon wafer were characterized by spectroscopic ellipsometry in the range 250–1000 nm using an M-2000UTM ellipsometer (J.A. Woollam Co., Lincoln, NE, USA). The incident angles were set to 50° and 60° and the acquisition time to 10 s. The data were fitted using the CompleteEASE software (version 5.20, J.A. Woollam Co.) representing the film as a Cauchy layer deposited onto the corresponding substrate.

Differential thermal analysis (DTA) and thermogravimetry (TG) were measured using the SDT Q600 instrument (TA Instruments, New Castle, DE, USA). Around 20–30 mg of powder materials with a size of 1–1.25 mm, obtained after drying the sol at 50 °C, were measured in air and Argon using a heating rate of 10 °C/min.

X-Ray diffraction (XRD) patterns of 80SiO_2_-20LaF_3_-0.5Er^3+^ self-supported layers and grazing incidence XRD (GI-XRD) of 80SiO_2_-20LaF_3_ thin films were obtained at the BM25B-SpLine beamline of the ESRF (European Synchrotron Radiation Facility, Grenoble, France) [39], using an X-ray wavelength of 0.619 Å. For GI-XRD measurements, an incidence angle of 0.5° was used. The crystal size, ϕ, was estimated using the Scherrer’s equation:

(1) $$\phi =\frac{0.94\lambda }{\cos\theta \;\sqrt{B_{m}^{2}-B_{i}^{2}}},$$

where λ is the wavelength, *B_m_* the full width half maximum of the peak and θ its diffraction angle. The instrumental broadening *B_i_* was also taken into account. The diffraction-peak parameters were fit using a pseudo Voigt function.

Rietveld Refinement was performed using the FullProf software (FullProf Suite July 2017) [40], and the LaF_3_ crystal fraction was estimated using NaF as the internal weight standard in a similar manner to that described previously [12]. X-ray powder data were collected over the range 20° ≤ 2θ ≤ 120° in a step width of 0.0289° and a counting time of 4s per step employing a Bruker D8 high-resolution diffractometer (BRUKER, Billerica, MA, USA) equipped with a solid-state rapid LynxEye detector, and monochromatic Cu Kα1 radiation.

High Resolution Transmission Electron Microscopy (HRTEM) was performed using a JEOL 2100 field emission gun (Akishima, Tokyo, Japan) operating at 200 kV with a point resolution of 0.19 nm. The samples were obtained by scratching the films or milling the self-supported layers and dispersing in EtOH. Then, one or two drops of suspensions were depositing on the scaled fragments onto carbon-coated copper grids. The solvent was removed after drying the copper grids under a UV lamp.

Er^3+^ L_3_ edge X-Ray absorption spectroscopy (XAS, Grenoble, France) were used to characterize the 80SiO_2_-20LaF_3_ 0.5Er^3+^ films treated at 150 °C for 1 h (xerogel) and at 550 °C for 1 min (GC) employing the BM25A-SpLine beamline (ESRF, Grenoble, France). XAS measurements were collected in fluorescence mode using a 13 element Si (Li) solid-state detector with the sample surface placed at an angle of 45° to the incident beam. The spectra represent an average of at least six scans; the XAS data were processed using the software ATHENA (Demeter 0.9.26) [41]. Both ErF_3_ and Er_2_O_3_ standards were also measured as references.

Electron Paramagnetic Resonance (EPR) spectra were obtained by a Bruker ESP 300E spectrometer (BRUKER) working in the X-band with field modulation of 100 kHz. The temperature was controlled with a continuous-flow helium cryostat ESR 900 (Oxford Instruments, Abingdon, UK) and the calibration of the resonance magnetic fields and microwave frequencies measured with an NMR Gauss-meter ER 035 M (BRUKER) and a frequency meter (Hewlett-Packard 5342A, Palo Alto, CA, USA), respectively. Self-supported layer samples (xerogel and GCs) doped with 0.1 and 0.5 Er^3+^ were milled and rolled in Teflon tape to perform the measurements.

The steady-state emission spectra were performed by exciting the 80SiO_2_-20LaF_3_ bulk samples and thin films doped with 0.1 and 0.5 Er^3+^ with a tunable Ti-sapphire ring laser (0.4 cm^−1^ linewidth) in the 770–920 nm spectral range. The fluorescence was analyzed with a 0.25 m monochromator, and the signal was detected by an extended IR Hamamatsu H10330A-75 photomultiplier (Hamamatsu, Shizuoka, Japan) and finally amplified by a standard lock-in technique. The sample temperature was varied between 9 and 300 K in a continuous flow cryostat.

Lifetime measurements were obtained by exciting the 80SiO_2_-20LaF_3_-0.1Er^3+^ (TEOS) bulk sample treated at 650 °C for 1 h with a Ti-sapphire laser pumped by a pulsed frequency-doubled Nd:YAG laser, 9 ns pulse width, (BM Industries, Paris, France), and detecting the emission with Hamamatsu H10330A-75 photomultiplier. Data were processed by a Tektronix oscilloscope (Tektronix Inc., Beaverton, OR, USA).

## 3. Results and Discussion

### 3.1. Self-supported Layers and Thin Films

**Figure 1:**
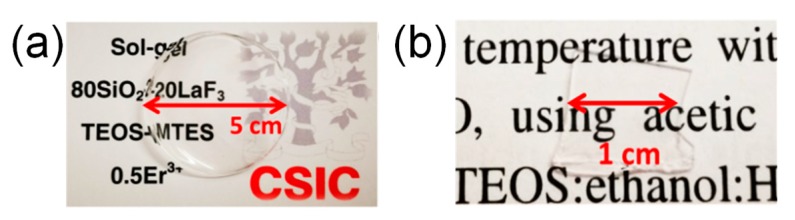
(**a**) Xerogel of composition 80SiO_2_-20LaF_3_- 0.5Er^3+^ (tetraethyl orthosilicate with methyl-trimethoxy-silane, TEOS-MTES) obtained at 150 °C for 24 h; (**b**) Oxyfluoride glass-ceramics (OxGC) of the same composition obtained at 550 °C for 1 min.

Crack-free and homogeneous self-supported layers were obtained before and after heat treatment of the doped and undoped 80SiO_2_-20LaF_3_ samples at 550 °C for 1 min, Figure 1.

**Figure 2:**
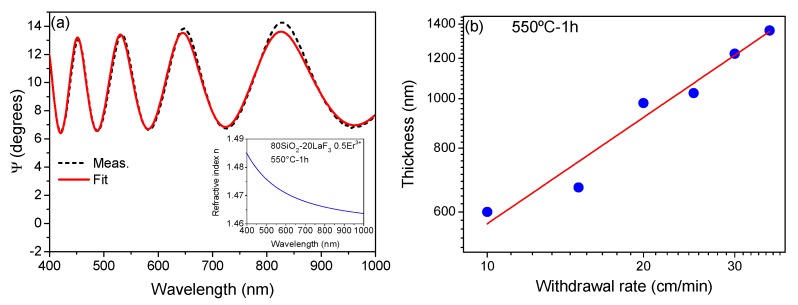
(**a**) Ellipsometric measurements and fit of 80SiO_2_-20LaF_3_-0.5Er^3+^ (TEOS-MTES) thin film treated at 550 °C for 1 min deposited onto silica substrate using a withdrawal rate of 35 cm/min. The inset shows the dispersion relation using the Cauchy model (**b**) Variation of thickness with the withdrawal rate.

Homogeneous and transparent films with good adhesion were obtained for all the withdrawal rates used. The good agreement between measurement and fitted data, Figure 2a, demonstrated the good quality of the films. The inset of Figure 2a shows the variation of the refractive index as a function of the wavelength in the transparent region of the sample. The corresponding thickness varies almost linearly with the withdrawal rate, up to a thickness of ~1.4 µm in a single deposition and using a withdrawal rate of 35 cm/min, Figure 2b. The addition of MTES allows the thickness of films to be increased, compared to when only TEOS is used. In particular, according to [42], the TEOS/MTES ratio 0.4/0.6 produces the highest critical thickness. Moreover, the addition of MTES improves the mechanical strength of the self-supported layers. Such behavior is associated with the presence of CH_3_ groups which allow the porosity to be increased in the silica network reducing the stress [42]. The amount of OH groups is also reduced when MTES is added to TEOS because the surfaces become more hydrophobic [42,43].

### 3.2. DTA and Crystallization

**Figure 3:**
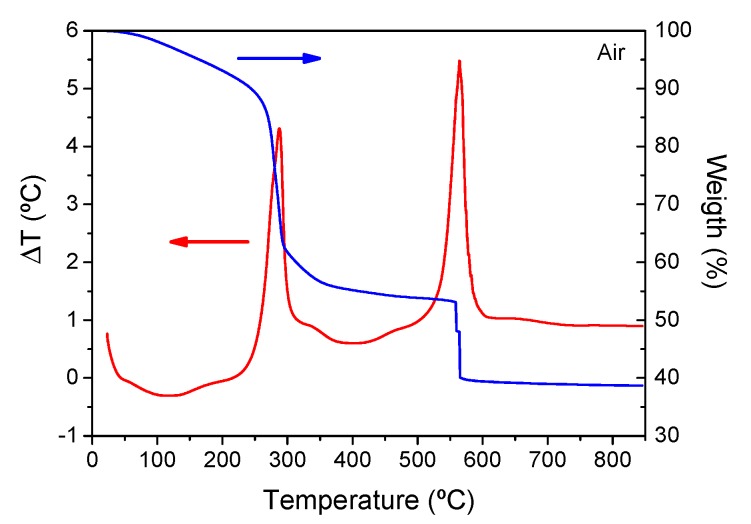
Differential thermal analysis (DTA, red) and thermogravimetry (TG, blue) curve for 80SiO_2_-20LaF_3_-0.5Er^3+^ (TEOS-MTES) bulk sample treated in the air with a heating rate of 10 °C/min.

Figure 3 shows a DTA curve for self-supported layers measured in air with a heating rate of 10 °C/min. The endothermic process at ~100 °C corresponds to water and solvent removal. The first sharp exothermic peak at ~280 °C is associated with LaF_3_ crystallization and the corresponding mass loss (~30%) is due to decomposition reactions, as studied elsewhere [44]. The sharpness of the crystallization peak means that the process occurs very rapidly; in contrast, the LaF_3_ crystallization peak in the melted glasses is much broader and less intense [12]. Moreover, the *T*_g_ of these samples is higher than 1000 °C, as reported in a previous paper [44], and the crystallization of SG OxGCs occurs at much lower temperature than the *T*_g_. The crystallization mechanism is, thus, clearly different with respect to that of MQ OxGCs [45,46,47,48,49]. The second exothermic process at ~575 °C, associated with oxidation of CH_3_ groups with a further mass loss of around 10%, does not appear in Ar atmosphere. Heat treatment at temperatures higher than that of CH_3_ elimination causes a sudden shrink of films and self-supported layers, leading to crack of the samples.

### 3.3. XRD, GI-XRD and Crystal Fraction

**Figure 4:**
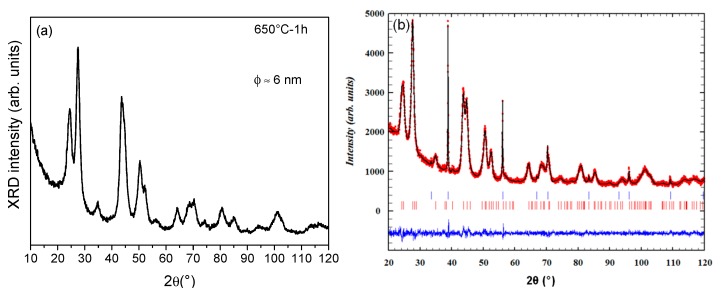
(**a**) X-Ray diffraction (XRD) of 80SiO_2_-20LaF_3_-01Er^3+^ (TEOS) self-supported layer treated at 650 °C for 1 h. (**b**) Observed (small red circles), calculated (continuous black line) and difference (continuous blue line at the bottom) XRD profiles of 80SiO_2_-20LaF_3_-0.5Er^3+^ (TEOS-MTES) self-supported layer treated at 550 °C mixed with 9 wt.% NaF as the internal standard. Bragg peaks of NaF and LaF_3_ are indicated by blue and red vertical bars, respectively.

The XRD of 80SiO_2_-20LaF_3_-0.1Er^3+^ (TEOS) self-supported layer treated at 650 °C for 1 h is shown in Figure 4a. As can be seen, only LaF_3_ NCs, with a crystal size of 6 nm, are obtained. On the other hand, XRD patterns of 80SiO_2_-20LaF_3_-0.5Er^3+^ (TEOS-MTES) self-supported layer treated at 550 °C for 1 min exhibited well-defined peaks consistent with the crystallization of LaF_3_ (JCPDS 00-032-0483) with a crystallite size of 9.0 ± 0.5 nm, demonstrating that only 1 min is sufficient to crystallize the phase, Figure 4b. The difference of the crystal size with respect to the TEOS sample treated at 650 °C is associated with the higher heating rate (10 °C/min) used to crystallize the samples prepared with TEOS-MTES. The crystallization mechanism of similar GCs was extensively studied in previous papers [44,50], demonstrating how the decomposition reaction of lanthanide fluoroacetates leads to precipitation of NCs once the crystallization temperature is reached. The crystallization mechanism of these materials is, therefore, completely different from that of oxyfluoride glasses by MQ in which the crystal growth is a diffusion-controlled process. Chemical bonds between fluorine and the glass matrix were observed using ^19^F Nuclear Magnetic Resonance (NMR) [44]. Instead, for the xerogel samples, the fluorine environment is exactly the same as in the precursor TFA acid; almost all fluorine is then found in the form of LaF_3_ after the crystallization process [44]. Furthermore, it was demonstrated that an increase of treatment time does not increase the crystal size or the crystal fraction of bulk samples; in contrast, crystal dissolution is observed for very long heat treatment times. Such results are clearly inconsistent with a diffusion-controlled process.

Quantitative Rietveld refinement of the LaF_3_ crystalline fraction with NaF as the internal standard was carried out for 80SiO_2_-20LaF_3_ 0.5Er^3+^ bulk GC treated at 550 °C for 1 min following the same procedure described in [49]. The observed diffraction pattern and the difference between observed and calculated diffraction patterns are shown in Figure 4b. Weight fractions (wt%) of 17.8% and 82.2% were determined for the LaF_3_ crystalline fraction and glassy phase, respectively. To our knowledge, this represents the highest crystal fraction reported in the literature for transparent OxGCs prepared by SG methods and it is the highest crystal fraction reported so far for LaF_3_-OxGCs prepared by both MQ and SG [38]. A small shift of the diffraction peaks towards higher 2θ was also observed for the Er^3+^-doped GC. This provides evidence of Er^3+^ incorporation, since the smaller Er^3+^ ions (1.17 Å) on the sites of the larger La^3+^ (1.31 Å) ion decrease the unit-cell volume (Table 1).

**Figure 5:**
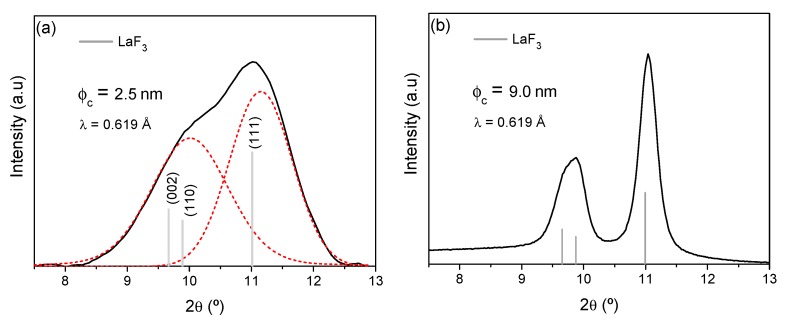
(**a**) Grazing incidence XRD (GI-XRD) of thin film and (**b**) XRD of the bulk sample of composition 80SiO_2_-20LaF_3_ 0.5Er^3+^ (TEOS-MTES) treated at 550 °C for 1 min. The crystal size is given together with the LaF_3_ reference shown with vertical bars (JCPDS 00-032-0483).

GI-XRD pattern of a thin film was obtained at the BM25B-SpLine beamline of the ESRF [39] and is shown in Figure 5a. The XRD of the bulk sample has also been acquired for comparison, Figure 5b. For the thin film, only a broad band, which could be deconvoluted into two peaks associated with LaF_3_ crystals (Figure 5a). In this case, the crystallite size was much smaller, ~2.5 ± 0.5 nm. The difference in crystallite size between bulk and film samples, is likely to be related to the synthesis and sintering conditions, since more diluted sols are necessary for film deposition with respect to bulk materials and during the sintering the coating densifies only by shrinkage normal to the substrate. Moreover, the fast evaporation of the solvent during the heat treatment may lead to the precipitation of such small NCs. This difference in crystal size between films and bulk-like materials has also been reported previously for similar systems, but a clear explanation is still necessary [52]. For comparison, Figure 5b shows the XRD of the bulk samples of the same composition treated under the same conditions.

### 3.4. High Resolution Transmission Electron Microscopy (HRTEM) and Nanostructure Characterization

**Figure 6:**
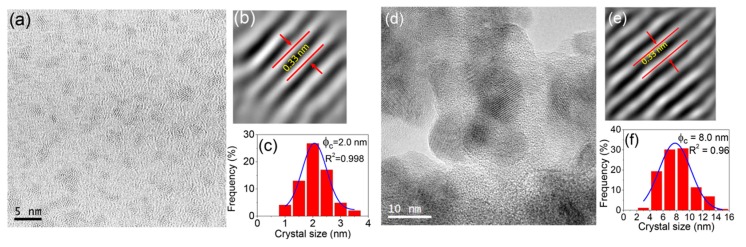
(**a**) High resolution transmission electron microscopy (HRTEM) micrograph, (**b**) lattice distance and (**c**) crystal-size distribution of 80SiO_2_-20LaF_3_ 0.5Er^3+^ thin film treated at 550 °C for 1 min. (**d**) HRTEM micrograph, (**e**) lattice distance and (**f**) crystal-size distribution of 80SiO_2_-20LaF_3_ 0.5Er^3+^ self-supported layer treated at 550 °C for 1 min.

HRTEM was used to reveal the lattice planes and crystal-size distribution of LaF_3_ crystals in the thin films (Figure 6a–c) and self-supported layers (Figure 6d–f) treated at 550 °C for 1 min. Small crystals are homogeneously distributed in the glass matrix. The crystal size is around 2.0 ± 0.5 nm and 8.0 ± 0.5 nm for films and self-supported layers, respectively, in agreement with the GI-XRD and XRD results. A similar structure is observed in both samples, consisting of separated crystals surrounded by the residual SiO_2_ matrix. Fourier Transformation of the (111) crystal planes of the LaF_3_ Tysonite phase was employed to estimate an interplanar distance of 0.33 nm [53]. These results demonstrate the absence of clusters even for such short heat treatment times and further support the hypothesis that the crystallization mechanism involves chemical decomposition followed by crystal precipitation.

### 3.5. XAS, EPR and RE Environment

A crucial aspect of RE-doped OxGCs concerns the distribution of dopants and the mechanism of incorporation into the fluoride NCs. In fact, RE ions are much more efficient when are embedded in low-phonon-energy fluoride crystals. Evidence of RE incorporation is often obtained by optical spectroscopy but sometimes only indirectly, unless specific selective excitation measurements are carried out. Here, the RE-ion environment and its role in transforming the xerogel to the GCs have been studied in detail using the local structural techniques XAS and EPR.

**Figure 7:**
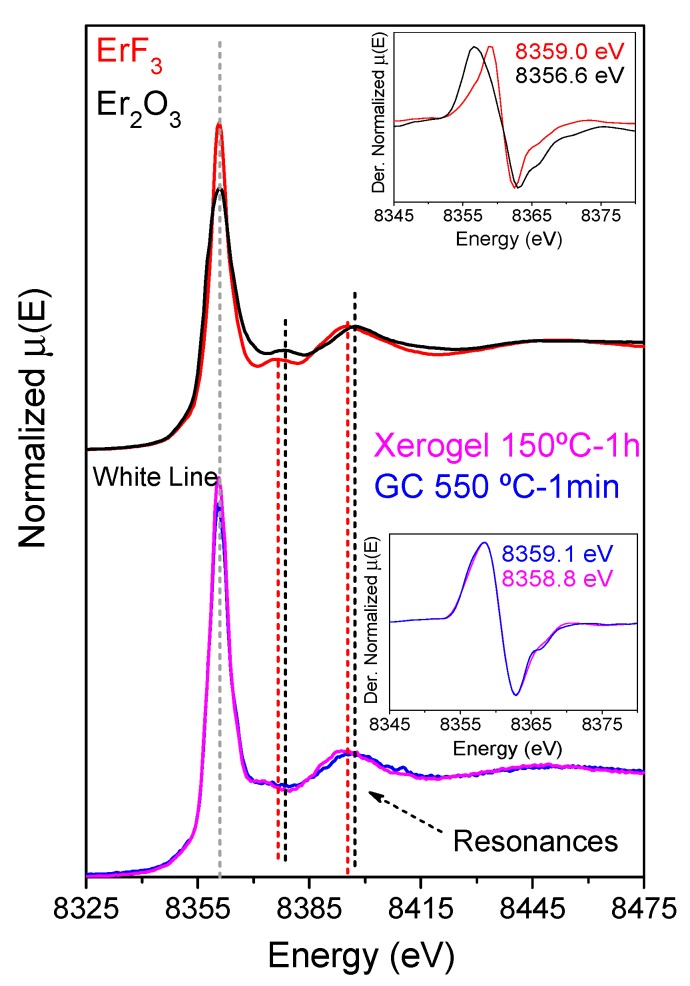
X-Ray absorption spectroscopy (XAS) spectra and derivatives (inset) of Er_2_O_3_ and ErF_3_ references in black and red, respectively (top), and of XAS spectra and derivatives (inset) of 80SiO_2_-20LaF_3_-0.5Er^3+^ (TEOS-MTES) xerogel and GC thin film treated at 550 °C for 1 min, in pink and blue, respectively (bottom). The grey line indicates the white line and the red and black lines indicate resonance positions for ErF_3_ and Er_2_O_3_, respectively.

XAS measurements of Er_2_O_3_ and ErF_3_ were used as a reference and compared to the results of thin films doped with 0.5Er^3+^, Figure 7. The presence of Er^3+^ is clearly indicated by the absorption edge centered around 8370 eV. However, small shifts of the edge are produced by different ligand fields. In general, for the same RE ion, the edge energy increases passing from oxides to fluorides, due to lesser hybridization of the chemical bonds in fluoride compounds attributable to a higher field strength [54,55,56]. In this case, the edge was obtained by the first derivative of the absorption spectrum and the edge shifts from 8356.6 to 8359 eV passing from Er_2_O_3_ to ErF_3_. More insights are obtained by a comparison of the post-edge region that contains information about the local structure. In particular, the X-Ray Absorption Near Edge Structure (XANES), shown in Figure 7 as red and black spectra, for ErF_3_ and Er_2_O_3_, respectively, show slight differences at the white line and at resonances of ~8375 and 8395 eV. The XANES spectra and derivative of the xerogel and OxGC thin film (bottom inset of Figure 7) exhibit an absorption edge centered at ~8359 eV, similar to pure ErF_3_. Hence, a fluorine-rich environment is already observed in the xerogel. Nevertheless, differences in the XANES region (8370–8415 eV) are observed compared to ErF_3_, which may indicate a change in the Er^3+^ environment for the xerogel and the OxGC samples. Fourier-Transform calculations indicate a narrowing of the first shell as the xerogel transforms to the OxGC, which is consistent with the development of a higher symmetry environment corresponding to the fluoride crystalline phase.

A linear combination of XANES spectra in the range 8325–8475 eV was performed for the OxGC sample using the standard ErF_3_ and Er_2_O_3_ materials. Employing the crystal fraction of LaF_3_ calculated by Rietveld refinement, approximate fractions of 91% and 9% were calculated for Er^3+^ in ErF_3_ and Er_2_O_3_-like environments, respectively. Thus, the Er^3+^ ions are likely to be situated near fluoride ions in the xerogel state, precipitating in fluoride-rich crystals after the heat treatment at 550 °C for 1 min. These results are explained considering that, in the starting sol, the La^3+^ and Er^3+^ ions are surrounded by TFA cations that are screened and impeded from bonding with Si atoms. Hence, in the xerogel sample, fluorine is still coordinated as in TFA acid [44] and Er^3+^ ions are principally coordinated by fluorine. During the heat treatment, decomposition promotes the precipitation of LaF_3_ crystals in which Er^3+^ ions become trapped. Other techniques, such as Energy Dispersive X-Ray Spectroscopy (EDXS), demonstrated an Eu enrichment in GdF_3_ NCs after just 1 min of treatment at 550 °C [50], further supporting the aforementioned conclusions.

Once the crystal fraction is obtained, an indication of the concentration of RE ions in LaF_3_ crystals may be obtained. Supposing that 91% of Er^3+^ ions are embedded in the crystal phase and that the crystalline fraction is ~18 wt% (XRD), the effective concentration of Er^3+^ ions in the crystal phase is around 6.5 mol%, representing an order of magnitude increase with respect to the nominal concentration (0.5 mol%). Similar values were obtained in previous studies of samples prepared by MQ, albeit with a lower fraction of RE ions incorporated in the crystal phase [57].

**Figure 8:**
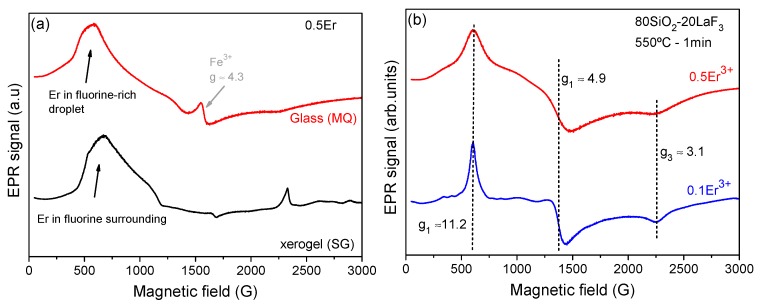
(**a**) Electron Paramagnetic Resonance (EPR) signals for the 80SiO_2_-20LaF_3_-0.5Er^3+^ (TEOS-MTES). xerogel and of an MQ glass doped with 0.5Er^3+^. (**b**) EPR signals of 80SiO_2_-20LaF_3_ (TEOS-MTES) GCs doped with 0.1 and 0.5Er^3+^ (mol%) treated at 550 °C for 1 min.

Further evidence supporting the mechanism for the incorporation RE ions is obtained by EPR of the self-supported layers. Figure 8 shows the EPR spectra of xerogel samples doped with 0.5 Er^3+^ (mol%) measured at 5 K. The EPR signal of a glass, prepared by MQ, containing 0.5 Er^3+^ in an amorphous fluorine-rich environment is also shown for comparison. Several resonances are observed for the xerogel samples, due to the presence of organic compounds which remain after heat treatment at 50 °C. Er^3+^ resonances are associated with the broad signals appearing in the range 500–1000 G. Other authors reported EPR measurements of Er^3+^ in oxyfluoride glasses showing the presence of broad signals at low magnetic fields (high *g* values) [58]. Moreover, such resonances disappear at room temperature (RT) and are only visible below 20 K, indicating that they are associated with Er^3+^ ions. Such broad signals are clearly related to an amorphous environment typical of glasses and liquids. The EPR signal of an oxyfluoride glass doped with 0.5 Er^3+^ prepared by MQ is also shown for comparison. The signal around 2300 G, observed only in the xerogel, is attributed to the presence of organic compounds. Interesting results are obtained for the GC samples, which display resonances associated with paramagnetic species with effective spin *S’* = 1/2 in rhombic or lower symmetry after just 1 min of heat treatment; the resonances appear at *g* values of *g*_1_ = 11.2; *g*_2_ = 4.9 and *g*_3_ = 3.1. On comparison with the EPR results obtained for an Er^3+^-doped LaF_3_ single crystal [59,60], it can be unambiguously concluded that the resonances in the GC samples are those of Er^3+^ in LaF_3_ NCs. Broader signals are observed for the GC doped with 0.5 Er^3+^ and associated with stronger relaxation processes, due to a moderately high effective concentration.

For the composition with a lower concentration of dopant, the hyperfine structure is only partially resolved, due to the interaction of the electrons with nuclear spins. The low intensity of the hyperfine structure is due to the quantity of Er isotope nuclei with *I* = 7/2 (~23%), while the remaining nuclei (77%) with *I* = 0 do not produce any hyperfine interaction [58,61,62]. For the composition doped with 0.5 Er^3+^, the hyperfine structure is too broad to be resolved but its presence may be discerned from the shape of the resonance at *g*_1_ = 11.2 which presents broader sidebands. Another interesting result is the absence of any further Er^3+^ resonance other than that of Er^3+^ in the LaF_3_ NCs.

These results, together with the previous discussion of XAS measurements, indicate that dopant incorporation occurs with crystal precipitation. In contrast, in melted glasses, residual contributions of RE ions in an amorphous environment are still observed even after the crystallization process, confirming that some RE ions remain in the glass matrix or in phase-separation droplets.

### 3.6. Photoluminescence (PL)

**Figure 9:**
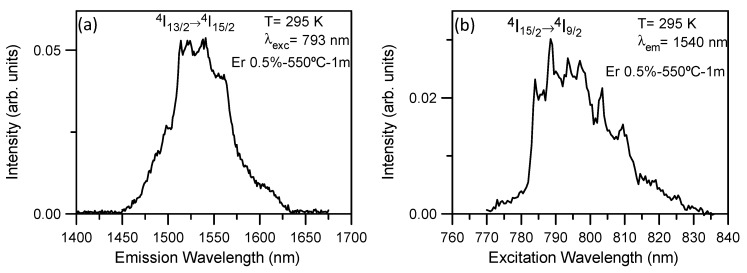
(**a**) Room temperature emission spectrum of 80SiO_2_-20LaF_3_-0.5Er^3+^ (TEOS-MTES) bulk sample heat treated at 550 °C for 1 min obtained on exciting at 793 nm. (**b**) Low-temperature excitation spectrum obtained at 1540 nm.

The near-infrared emission spectra corresponding to the ^4^I_13/2_→^4^I_15/2_ transition for 80SiO_2_-20LaF_3_-0.5Er^3+^ (TEOS-MTES) self-supported layer heat treated at 550 °C for 1 min were obtained at room temperature and at 9 K by exciting at 793 nm in the ^4^I_15/2_→^4^I_9/2_ absorption band. After excitation of this level, the next lower levels are populated by multiphonon relaxations. The room temperature emission spectrum (Figure 9a) shows a series of peaks with a maximum of around 1540 nm. Figure 9b shows the excitation spectrum corresponding to the ^4^I_15/2_→^4^I_9/2_ transition obtained by collecting the luminescence spectrum at 1540 nm, revealing well defined peaks which suggest the partial incorporation of Er^3+^ ions in the LaF_3_ NCs.

**Figure 10:**
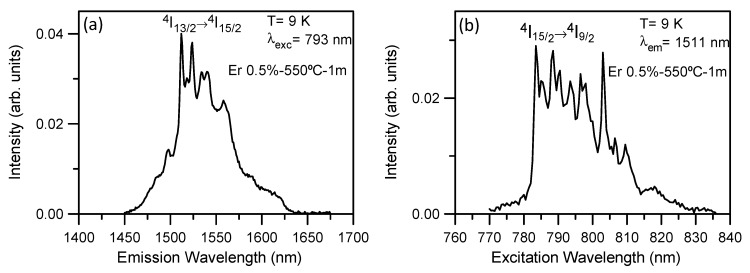
(**a**) Low temperature emission spectrum of 80SiO_2_-20LaF_3_-0.5Er^3+^ (TEOS-MTES) bulk sample heat treated at 550 °C for 1 min on exciting at 793 nm. (**b**) Low temperature excitation spectrum obtained at 1511 nm.

The incorporation of Er^3+^ ions into LaF_3_ NCs is unambiguously confirmed by measurements performed at low temperature. Figure 10 shows the emission and excitation spectra, respectively, obtained at 9 K. The emission spectrum shows sharp peaks superimposed on a broad band. The excitation spectrum obtained by collecting the luminescence at the emission maximum also shows very well defined peaks. The features exhibited by the low-temperature spectra confirm the partial incorporation of Er^3+^ ions in the NCs and provide evidence of the crystalline and amorphous Er^3+^ environments.

**Figure 11:**
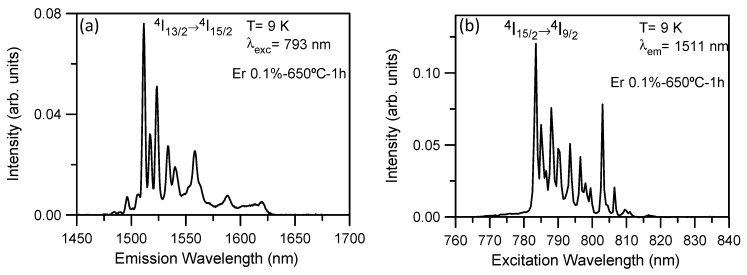
(**a**) Low-temperature emission spectrum of the 80SiO_2_-20LaF3-0.1Er^3+^ (TEOS) bulk sample heat treated at 650 °C for 1 h on exciting at 793 nm. (**b**) Low temperature excitation spectrum obtained at 1511 nm.

Measurements performed for the 80SiO_2_-20LaF_3_-0.1Er^3+^ (TEOS) bulk sample heat treated at 650 °C for 1 h suggest that Er^3+^ ions are mainly incorporated in LaF_3_ NCs. As an example, Figure 11a shows the low-temperature emission spectrum obtained on exciting at 793 nm. In this case, sharp and well-defined peaks are observed. Moreover, the excitation spectrum obtained by collecting the luminescence at the emission maximum corresponds to a crystalline environment for Er^3+^ ions. Similar emission spectra are obtained by exciting different peaks in the spectrum.

These results confirm that, in the 80SiO_2_-20LaF_3_-0.1Er^3+^ (TEOS) bulk sample, the Er^3+^ ions are mainly located in the LaF_3_ NCs. Although, the emission and excitation spectra of the 80SiO_2_-20LaF_3_-0.5Er^3+^(TEOS/MTES) bulk sample also show well-defined peaks, they are much broader and less well resolved than those of the less concentrated sample, suggesting the presence of both crystalline and amorphous Er^3+^ environments in the 0.5% Er^3+^-doped 80SiO_2_-20LaF_3_ sample.

**Figure 12:**
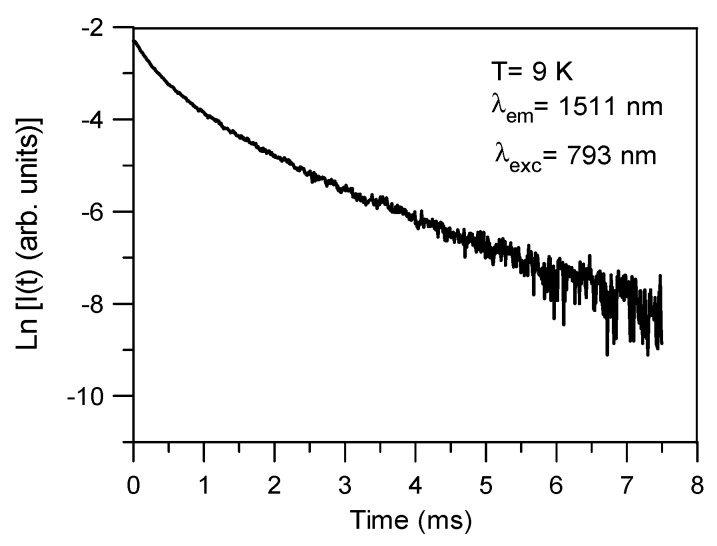
Semi-logarithmic plot of the fluorescence decay of the ^4^I_13/2_ level for the 80SiO_2_-20LaF_3_-0.1Er^3+^ (TEOS) bulk sample doped with 0.1% Er^3+^ obtained by exciting at 793 nm and collecting the luminescence at 1511 nm.

Another important spectroscopic parameter to characterize the luminescent behavior of Er^3+^ ions is the decay time of the emitting level. The lifetime of the ^4^I_13/2_ level was obtained at 9K by exciting at 793 nm and collecting the luminescence at 1511 nm. The decay for the 80SiO_2_-20LaF_3_-0.1Er^3+^ (TEOS) bulk sample (Figure 12) deviates from a single exponential function and the lifetime is unexpectedly short. The average lifetime, calculated by:

(2) $$\langle \tau \rangle =\frac{\int_{0}^{\infty }tI\left(t\right)dt}{\int_{0}^{\infty }I\left(t\right)dt},$$

where *I*(*t*) represents the luminescence intensity at time *t* corrected for the background, is around 0.79 ms. This lifetime is nearly one order of magnitude shorter than those observed in bulk samples prepared by MQ [13]. Similar behavior has also been observed in Nd^3+^-doped 80SiO_2_-20LaF_3_ thin films, and attributed to the fact that the effective RE^3+^-ion concentration in the NCs differs by up to an order of magnitude from the theoretical (or nominal) value [12,52].

**Figure 13:**
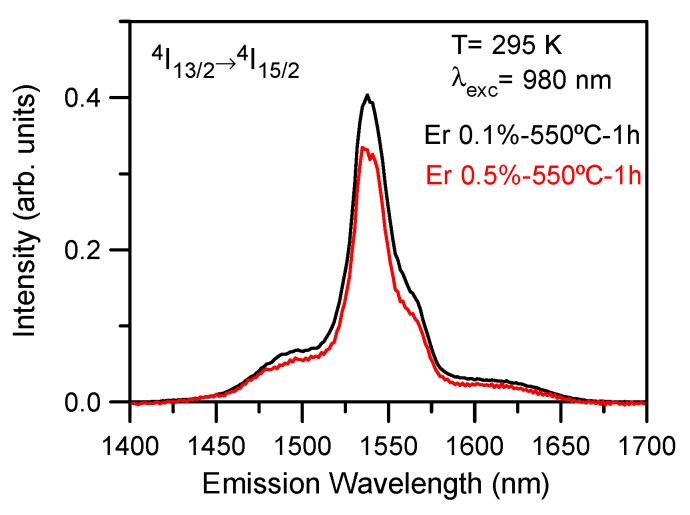
Room temperature emission spectrum of 80SiO_2_-20LaF_3_ (TEOS-MTES) thin films doped with 0.1% (black line) and 0.5% (red line) Er^3+^ heat treated at 550 °C for 1 h obtained on exciting at 980 nm.

The room temperature emission spectra of 80SiO_2_-20LaF_3_ (TEOS-MTES) thin films doped with 0.1 and 0.5 Er^3+^ heat treated at 550 °C for 1 h corresponding to the ^4^I_13/2_→^4^I_15/2_ transition obtained by exciting at 980 nm reveal a glass-like behavior. Figure 13 shows the emission spectra for thin films doped with 0.1 and 0.5% Er^3+^. In both samples the spectrum shows an unresolved structure with a maximum at around 1540 nm which indicates the absence of a crystalline environment for Er^3+^ ions.

## 4. Conclusions

For the first time the preparation of transparent oxyfluoride glass ceramics of composition 80SiO_2_-20LaF_3_ containing Er^3+^-dopant have been prepared by sol-gel using TEOS and MTES as silica precursors. XRD and HRTEM showed the precipitation of LaF_3_ nanocrystals, with a size of 9 and 3 nm, after heat treatment at 550 °C for 1 min for bulk and thin film, respectively. Rietveld refinement indicated a LaF_3_ crystal fraction of ~18 wt% for the bulk glass ceramic. To our knowledge, this represents the highest crystal fraction reported in the literature for transparent oxyfluoride glass ceramics prepared by both melt-quenching and sol-gel methods. XAS and EPR shown a fluorine-rich amorphous environment in the xerogel and confirmed the presence of Er^3+^ in the LaF_3_ nanocrystals after crystallization, with a concentration around one order of magnitude higher than the nominal value. No XAS and EPR results have previously been reported for RE-doped oxyfluoride glass-ceramics prepared by sol-gel. The incorporation of Er^3+^ ions into LaF_3_ nanocrystals was unambiguously confirmed by measurements performed at low temperature. For doping levels higher than 0.1 mol%, contributions from crystalline and amorphous environments are observed. A clear difference is found between the emission spectra of thin films and bulk samples, likely related to the different crystal size.

## Figures and Tables

**Table 1:** Lattice constants of 80SiO_2_-20LaF_3_-0.5Er^3+^ (TEOS-MTES) glass-ceramic bulk sample treated at 550 °C for 1 min.

Sample	a (Å)	c (Å)	Unit Cell Volume (Å^3^)
Undoped LaF_3_ [JCPDS]^1^	7.187	7.350	328
Er^3+^ doped GCs 550°C 1 m	7.16 ± 0.01	7.32 ± 0.01	325 ± 1

^1^ JCPDS cards 00-032-0483. Ionic sizes in n-fold coordination for hexagonal phase: 1.31 Å for La^3+^ and 1.17 Å for Er^3+^ [51].
